# Sepsis associated with acute lung injury over the period 2012–2021: a bibliometric analysis

**DOI:** 10.3389/fphys.2023.1079736

**Published:** 2023-06-15

**Authors:** Guibin Liang, Wenhua Wang, Zhihui He

**Affiliations:** Department of Critical Care Medicine, The Third Xiangya Hospital, Central South University, Changsha, Hunan, China

**Keywords:** sepsis, acute lung injury, bibliometric analysis, NF-κB, programmed cell death

## Abstract

**Background:** Sepsis associated with acute lung injury (ALI) is a common acute and severe disease with severe socioeconomic burden. The aim of this study is to explore the literatures of sepsis associated with ALI from a bibliometric perspective.

**Methods:** Articles and reviews related to sepsis associated with ALI published from 2012 to 2021 in the Web of Science Core Collection were retrieved. Countries, affiliations, journals, authors, references, co-citation and keyword analysis in this field were visually analyzed using WOS citation reports, bibliometric.com, CtieSpace and VOSviewer software.

**Results:** Over the last decade (2012–2021), marked progress has been made in the area of sepsis associated with ALI research. 836 papers were enrolled in this study. China accounts for the most contributors. Articles from the United States has the highest average cited. Shanghai Jiao Tong University, University of California System and Huazhong University of Science Technology were the main contributing institutions. Articles in International Immunopharmacology, Inflammation, Shock and Critical Care were cited the most. Matthay MA and Ware LB were the main contributors to this field. Inflammation and NF-κB have always been the focus of sepsis associated with ALI related research, and programmed cell death (including apoptosis, necroptosis and pyroptosis) may be the important direction of future research.

**Conclusion:** Research on the sepsis associated with ALI is flourishing. The research on programmed cell death is a hot spot and may be a promising research field in the coming years.

## 1 Introduction

Sepsis is a life-threatening organ dysfunction caused by the dysregulation of the body’s response to infection (Singer et al., 2016). The global study of adult sepsis hospitalized patients showed that the overall mortality rate of sepsis patients was 26.7%, while the mortality rate of sepsis patients in intensive care unit (ICU) was 41.9% (Fleischmann-Struzek et al., 2020). According to studies in China, the annual medical cost of 230,000 septic patients treated in ICU is about $4.6 billion (Xie et al., 2020). Sepsis has become an important global public health problem. Acute lung injury (ALI) is one of the most common complications of sepsis. Study has shown that 6%–42% of ALI is caused by sepsis (Sessler et al., 1996). ALI caused by sepsis refers to acute hypoxic respiratory failure caused by sepsis, which is secondary to alveolar injury caused by dysregulation of inflammatory response. The pathophysiology of ALI is mainly manifested by severe inflammatory injury of alveolar capillary barrier, depletion of pulmonary surfactant, reduction of effective ventilation lung tissue, and reduction of lung compliance (Ranieri et al., 2012; Matthay et al., 2019). Since ALI was first described in 1967, people’s understanding and definition of its clinicopathology have been constantly updated. Nowadays, the clinical diagnosis and treatment of ALI have always followed the Berlin definition in 2012 (Ranieri et al., 2012). Since ALI is a complex and clinically heterogeneous syndrome, the effective treatment strategies that can reach consensus today are limited to protective ventilation therapy (auxiliary neuromuscular blockers and prone ventilation), while other anti-inflammatory drugs [e.g., glucocorticoids, macrophage colony stimulating factor (GM-CSF), statins, aspirin] and drugs to improve lung function (β receptor agonists, nitric oxide inhalation) did not significantly reduce the mortality of ALI (Bellani et al., 2016). ALI associated with sepsis involves multiple molecular mechanisms. Consequently, it is significant to quantitatively analyze the *status quo*, focus areas, and future prospects related to ALI associated with sepsis.

Bibliometrics is an interdisciplinary science that uses mathematical and statistical methods to study literature and bibliometrics features (Wu et al., 2022). Bibliometrics uses mathematical methods to break through the limitations of time and space, so that researchers can fully understand the current situation, hot spots and trends of a research field. Currently, bibliometric analysis has been used in many fields, including atherosclerosis, Parkinson’s disease, gout and liver transplantation (Jiang et al., 2022; Li et al., 2022; Wang et al., 2022; Wen et al., 2022). To date, there is no literature about bibliometrics analysis of sepsis associated with ALI. To fill this gap, this study analyzed the characteristics and trends of publications related to sepsis associated with ALI through bibliometrics.

## 2 Materials and methods

### 2.1 Data sources and search strategy

The search was conducted using the Web of Science (WOS) Core Collection database. All the literature was retrieved in WOS. The search terms were TS= (sepsis OR endotoxemia) AND TS= ((acute lung injury) OR (ALI) OR (acute respiratory distress syndrome) OR (respiratory distress syndrome) OR (ARDS)). The language is limited to English, and the document types are limited to article or review. For the time span, we chose the 10 years between 2012 and 2021. In total, 4,790 articles were initially identified in our study. Two reviewers independently screened the titles and abstracts to exclude literature which unrelated to sepsis associated with ALI. After the full-text reading, 836 articles that exclusively addressed the topic of sepsis associated with ALI were included. Since the data of this study is from WOS, which is an open database, there are no ethical issues related to access in this study.

### 2.2 Bibliometric analyzing

**FIGURE 1:**
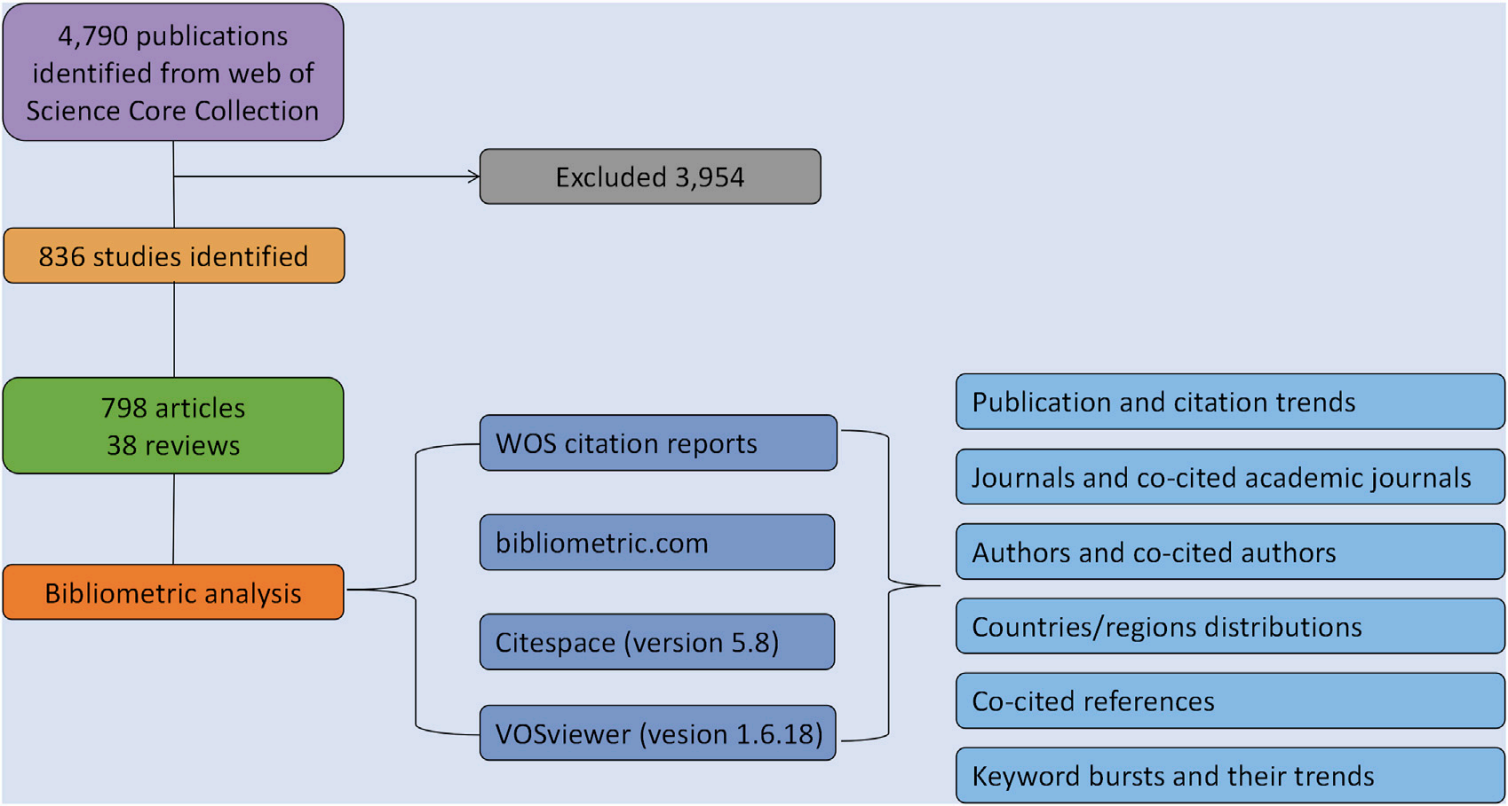
Detailed procedures of the enrolment and analysis.

“Citation Report” function of the web science was employed to evaluate the citation rates and the H-index. The “bibliometric.com” is a website for online bibliometric analysis (https://bibliometric.com/). CiteSpace (Vesion 5.8) is a widely used visual analysis software (Chen, 2004). VOSviewer (Vesion 1.16.18) is a software for plotting maps based on network data (van Eck and Waltman, 2010). We used WOS citation reports, bibliometric.com, CtieSpace and VOSviewer software for bibliometric analysis. Detailed procedures of the enrolment and analysis are illustrated in Figure 1.

## 3 Results

### 3.1 The research trends

**FIGURE 2:**
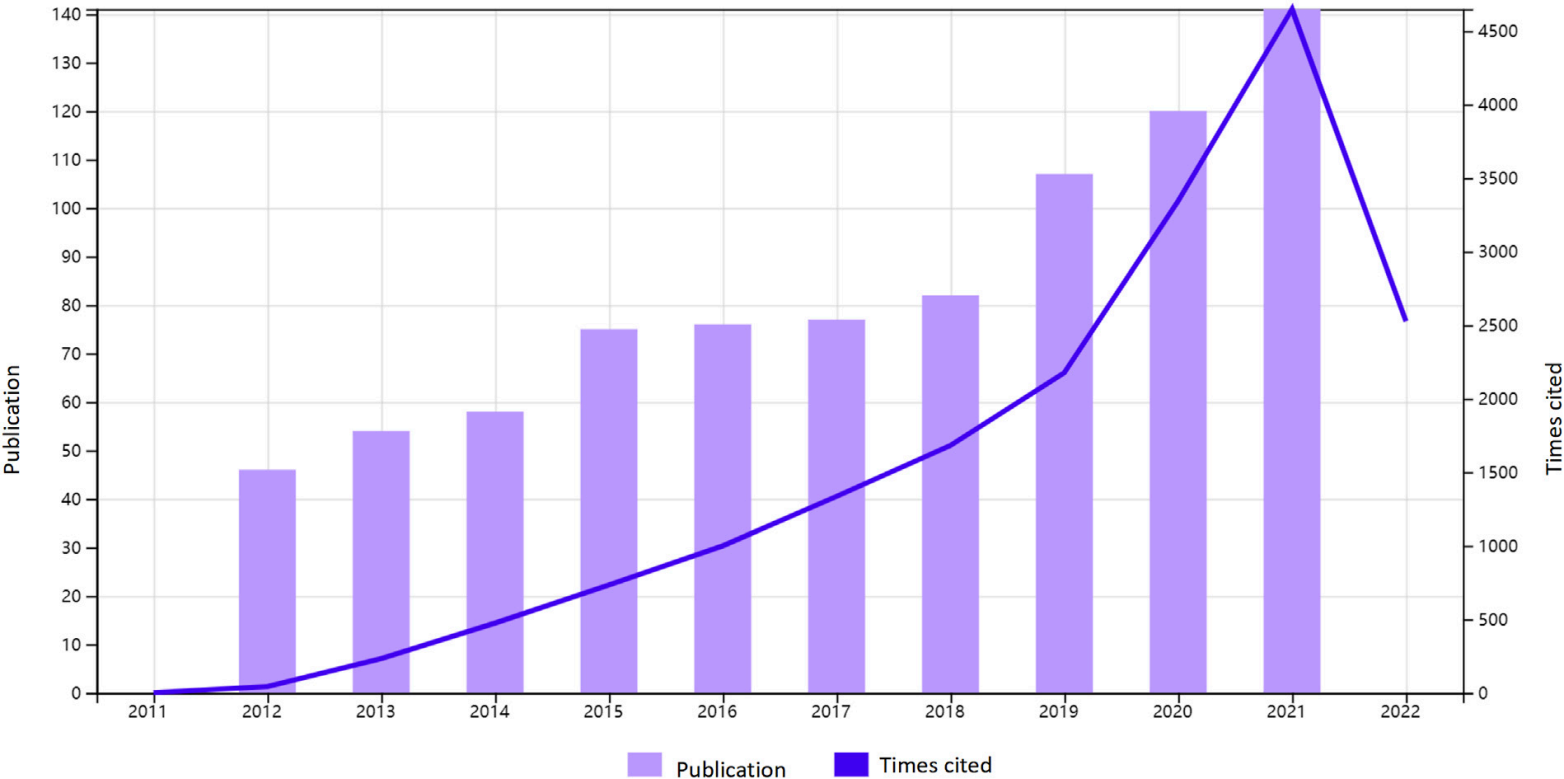
The number of publications by year over the past 10 years.

We collected 836 publications from the Web of Science core collection published in 312 journals over the period 2012–2021. The number of publications related to sepsis associated with ALI by year was presented in Figure 2. In total, 836 publications have been cited 13,491 times, and the average number of citations per publication is 21.76 times.

### 3.2 Analysis of the top 10 countries

**FIGURE 3:**
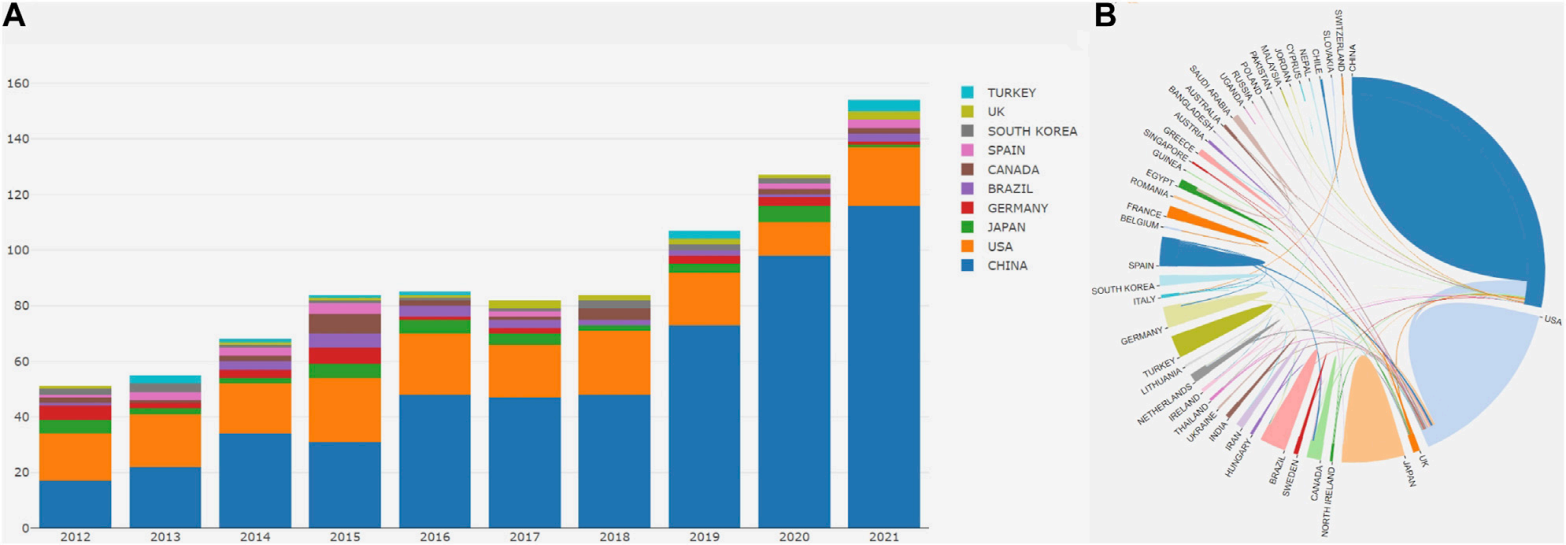
The number of publications by country **(A)** The total of publications by each country each year; **(B)** The cooperative relationship between each country.

The top 10 most productive countries were shown in Table 1. Table 1 shows the total citations, average number of citations per publication, H-index and publications/population (per million people) of the top 10 countries. Among the top 10 countries, China ranked first in the sum of total publications (534), cited times (9,331) and H-index (43), but only ranked seventh in the average cited times of a single article (17.47). In terms of the total publications, the United States ranked second. However, the United States ranked first in the average number of citations per publication (38.31). It was followed by Japan, Germany, Brazil, Canada, Spain, South Korea, United Kingdom and Turkey. Canada ranked first in publications/population (per million people) (0.61). Figure 3A showed the total of publications by each country each year, and Figure 3B showed the cooperative relationship between each country. The results showed the greatest cooperation between China and the United States, and the United States cooperated with other countries more broadly.

**TABLE 1:** Publications in the 10 most productive countries/regions.

No.	Country	Counts (%)	Total cites	Average cites	H-index	Publications/population (per million people)
1	China	534 (59.532%)	9,331	17.47	43	0.41
2	United States of America	194 (21.628%)	7,432	38.31	42	0.58
3	Japan	35 (3.902%)	724	20.69	16	0.27
4	Germany	27 (3.010%)	671	24.85	15	0.33
5	Brazil	24 (2.676%)	247	10.29	11	0.11
6	Canada	23 (2.564%)	639	27.78	13	0.61
7	Spain	18 (2.007%)	379	21.06	13	0.38
8	South Korea	16 (1.784%)	256	16	10	0.31
9	United Kingdom	13 (1.449%)	398	30.62	8	0.19
10	Turkey	13 (1.449%)	257	19.77	7	0.15

### 3.3 Analysis of the top 10 affiliations

The top 10 productive affiliations were shown in Table 2. Shanghai Jiao Tong University had the highest the sum of total publications (32), followed by University of California System (25), Huazhong University of Science Technology (25), University of Pennsylvania (21), Wenzhou Medical University (21), Wuhan University (21), Nanjing Medical University (20), China Medical University (19), Tongji University (19) and Harvard University (17). University of California System ranked first in terms of the average number of citations per publication (71.24), the highest cited times (1,781) and H-index (17). Nanjing Medical University ranked first in publications/students (per thousand people) (1.33). Although the total publications of Nanjing Medical University in China were relatively high, their average number of citations per publication was far behind other high-yield universities. It may be related to the low quality of the published articles and the relatively obscure journals.

**TABLE 2:** The top 10 productive affiliations.

No.	Affiliations	Country	Counts	Total cites	Average cites	H-index	Publications/students (per thousand people)
1	Shanghai Jiao Tong University	China	32	858	26.81	12	0.71
2	University of California System	United States of America	25	1781	71.24	17	0.05
3	Huazhong University of Science Technology	China	25	352	14.08	12	0.42
4	University of Pennsylvania	United States of America	21	468	22.29	13	1.05
5	Wenzhou Medical University	China	21	379	18.05	11	1.31
6	Wuhan University	China	21	296	14.1	10	0.36
7	Nanjing Medical University	China	20	153	7.65	7	1.33
8	China Medical University	China	19	438	23.05	13	1.06
9	Tongji University	China	19	406	21.37	11	0.54
10	Harvard University	United States of America	17	1,021	60.06	14	0.55

### 3.4 Analysis of the top 10 journals

The top 10 most active journals were shown in Table 3. International Immunopharmacology (IF = 5.714, 2021) was the most active journal in sepsis associated with ALI research, with 35 articles, followed by Inflammation (IF = 4.657, 2021), with 27 articles; Shock (IF = 3.533, 2021), with 26 articles; Critical Care (IF = 19.334, 2021), with 21 articles; American Journal of Physiology Lung Cellular and Molecular Physiology (IF = 6.011, 2021), with 15 articles; Journal of Surgical Research (IF = 2.417, 2021), with 15 articles; Molecular Medicine Reports (IF = 3.423, 2021), with 15 articles; Scientific Reports (IF = 4.996, 2021), with 15 articles; Biomedicine Pharmacotherapy (IF = 7.419, 2021), with 12 articles and Critical Care Medicine (IF = 9.296, 2021), with 12 articles. Among the top 10 journals, International Immunopharmacology published the most sepsis associated with ALI related articles. International Immunopharmacology was an interdisciplinary journal devoted to the publication of original scientific papers interrelating immunology and pharmacology. Additionally, Critical Care (836 citations) had the most total citations. Inflammation (H-index of 18) ranked first in H-index. Critical Care aimed to improve the care of critically ill patients by acquiring, discussing, distributing, and promoting evidence-based information relevant to intensivists. In addition, American Journal of Physiology Lung Cellular and Molecular Physiology had relatively high average number of citations per publication (39.33 average citations). The American Journal of Physiology Lung Cellular and Molecular Physiology published original research covering the broad scope of molecular, cellular, and integrative aspects of normal and abnormal function of cells and components of the respiratory system.

**TABLE 3:** The top 10 most active journals.

No.	Journal	Counts	Total cites	Average cite	H-index	If (2021)	JCR	Scope
1	International Immunopharmacology	35	750	21.43	16	5.714	Q1/Q2	An interdisciplinary journal devoted to the publication of original scientific papers interrelating immunology and pharmacology
2	Inflammation	27	801	29.67	18	4.657	Q3	The latest international advances in experimental and clinical research on the physiology, biochemistry, cell biology, and pharmacology of inflammation
3	Shock	26	739	28.42	16	3.533	Q2/Q3	A vehicle for timely publication in the areas of basic and clinical studies of shock, trauma, sepsis, inflammation, ischemia, and related pathobiological states, with particular emphasis on the biologic mechanisms that determine the response to such injury
4	Critical Care	21	836	39.81	16	19.334	Q1	Aims to improve the care of critically ill patients by acquiring, discussing, distributing, and promoting evidence-based information relevant to intensivists
5	American Journal of Physiology Lung Cellular and Molecular Physiology	15	590	39.33	14	6.011	Q1/Q2	Original research covering the broad scope of molecular, cellular, and integrative aspects of normal and abnormal function of cells and components of the respiratory system
6	Journal of Surgical Research	15	291	19.4	11	2.417	Q3	Original articles concerned with clinical and laboratory investigations relevant to surgical practice and teaching
7	Molecular Medicine Reports	15	344	22.93	9	3.423	Q3	Studies devoted to molecular medicine, underscoring aspects including pharmacology, pathology, genetics, neurosciences, infectious diseases, molecular cardiology and molecular surgery
8	Scientific Reports	15	471	31.4	12	4.996	Q2	All areas of the natural sciences, psychology, medicine and engineering
9	Biomedicine Pharmacotherapy	12	282	23.5	10	7.419	Q1	A multidisciplinary journal which publishes full-length, original research reports, reviews, and preliminary communications or letters to the editor which fall within the general scope of clinical and basic medicine and pharmacology
10	Critical Care Medicine	12	239	19.92	10	9.296	Q1	Original articles on significant work in critical care medicine

### 3.5 Analysis of the top 10 authors

The top 10 authors with the most publications were shown in Table 4. 4,366 authors contributed to sepsis associated with ALI related publications. Matthay MA was the most productive which published 11 articles (1,398 citations and 127.9 average citations), followed by Ware LB (11 articles, 534 citations and 48.55 average citations), Calfee, CS (9 articles, 625 citations and 69.44 average citations), Meyer, NJ (9 articles, 313 citations and 34.78 average citations), Villar, J (8 articles, 147 citations and 18.38 average citations), Christie, JD (7 articles, 294 citations and 42 average citations), Reilly, JP (7 articles, 208 citations and 29.71 average citations), Shi Jia (6 articles, 133 citations and 22.17 average citations), Zhang Yuan (6 articles, 133 citations and 22.17 average citations) and Flores, C (6 articles, 119 citations and 19.83 average citations). In addition, most of the top 10 authors were from the United States of America (6), China (2) or Spain (2).

**TABLE 4:** The top 10 authors with the most publications.

No.	Author	Country	Count	Total cites	Average cites
1	Matthay MA	United States of America	11	1,398	127.9
2	Ware LB	United States of America	11	534	48.55
3	Calfee CS	United States of America	9	625	69.44
4	Meyer NJ	United States of America	9	313	34.78
5	Villar J	Spain	8	147	18.38
6	Christie JD	United States of America	7	294	42
7	Reilly JP	United States of America	7	208	29.71
8	Shi Jia	China	6	133	22.17
9	Zhang Yuan	China	6	133	22.17
10	Flores C	Spain	6	119	19.83

### 3.6 Analysis of the top 10 most cited publications

Table 5 shows the 10 most cited articles, and the range of citations was from 158 to 439. “The pulmonary endothelial glycocalyx regulates neutrophil adhesion and lung injury during experimental sepsis (Schmidt et al., 2012)” published by Schmidt EP et al. (2012) and had 439 citations; “Human Mesenchymal Stem Cell Microvesicles for Treatment of *Escherichia coli* Endotoxin-Induced Acute Lung Injury in Mice (Zhu et al., 2014)”, published by Zhu YG et al. (2014), which was with 426 citations; and the third with 373 citations was the “Effect of Vitamin C Infusion on Organ Failure and Biomarkers of Inflammation and Vascular Injury in Patients With Sepsis and Severe Acute Respiratory Failure: The CITRIS-ALI Randomized Clinical Trial (Fowler et al., 2019)” published by Fowler AA third. et al. (2019).

**TABLE 5:** The top 10 most cited publications.

No.	Title	Author	Journal	Year	Type	If (2021)	Times cited
1	The pulmonary endothelial glycocalyx regulates neutrophil adhesion and lung injury during experimental sepsis	Schmidt EP et al	Nature Medicine	2012	Article	87.241	439
2	Human Mesenchymal Stem Cell Microvesicles for Treatment of *Escherichia coli* Endotoxin-Induced Acute Lung Injury in Mice	Zhu YG et al	Stem Cells	2014	Article	5.845	426
3	Effect of Vitamin C Infusion on Organ Failure and Biomarkers of Inflammation and Vascular Injury in Patients With Sepsis and Severe Acute Respiratory Failure: The CITRIS-ALI Randomized Clinical Trial	Fowler AA 3rd et al	JAMA-Journal of The American Medical Association	2019	Article	157.335	373
4	Rosuvastatin for Sepsis-Associated Acute Respiratory Distress Syndrome	National Heart, Lung, and Blood Institute ARDS Clinical Trials Network	New England Journal of Medicine	2014	Article	176.079	331
5	Enrichment of the lung microbiome with gut bacteria in sepsis and the acute respiratory distress syndrome	Dickson RP et al	Nature Microbiology	2016	Article	30.964	240
6	Caspase-11-mediated endothelial pyroptosis underlies endotoxemia-induced lung injury	Cheng KT et al	Journal of Clinical Investigation	2017	Article	19.456	191
7	Conservative fluid management or deresuscitation for patients with sepsis or acute respiratory distress syndrome following the resuscitation phase of critical illness: a systematic review and meta-analysis	Silversides JA et al	Intensive Care Medicine	2017	Review	41.787	181
8	Toward Smarter Lumping and Smarter Splitting: Rethinking Strategies for Sepsis and Acute Respiratory Distress Syndrome Clinical Trial Design	Prescott HC et al	American Journal of Respiratory and Critical Care Medicine	2016	Article	30.582	176
9	Anti-Inflammatory Effects of Apigenin in Lipopolysaccharide-Induced Inflammatory in Acute Lung Injury by Suppressing COX-2 and NF-kB Pathway	Wang J et al	Inflammation	2014	Article	4.657	173
10	Mesenchymal stem cells: mechanisms of potential therapeutic benefit in ARDS and sepsis	Walter J et al	Lancet Respiratory Medicine	2014	Review	102.642	158

### 3.7 Co-citation analysis

#### 3.7.1 Journals

**FIGURE 4:**
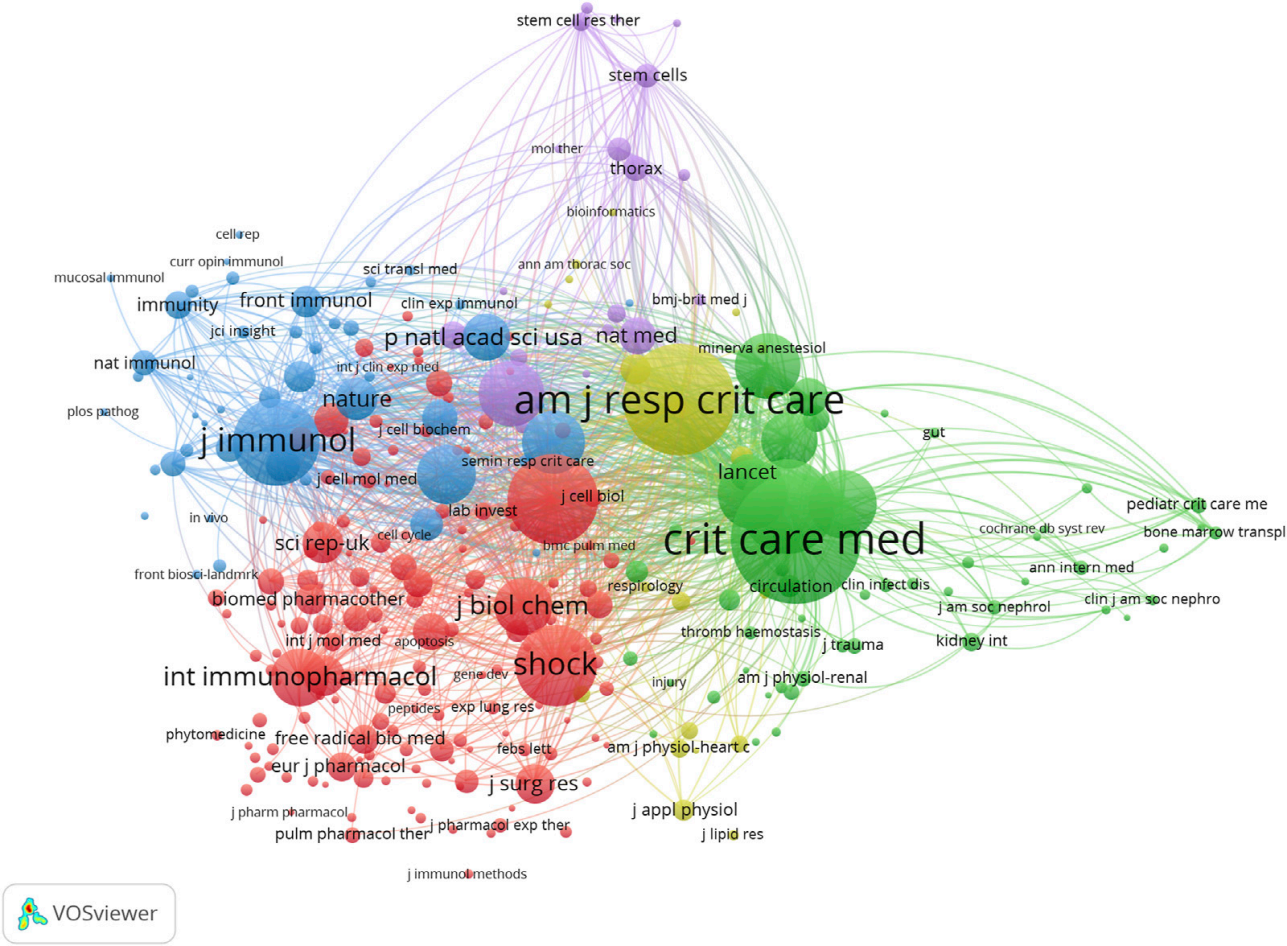
The mapping on journals of sepsis associated with ALI. The relationship between 288 identified journals (the minimum number of citations of journals exceeds 20) and the total link strength.

Figure 4 shows the relationship between 288 identified journals (the minimum number of citations of journals exceeds 20) and the total link strength using VOSviewer. As presented in Table 6, the top 10 journals by total link strength were as follows: Critical Care Medicine (total link strength = 52,752 times), American Journal of Respiratory and Critical Care Medicine (46,606 times), New England Journal of Medicine (6,234 times), Journal of immunology (34,933 times), American Journal of Physiology-lung Cellular and Molecular Physiology (33,680 times), Shock (25,489 times), Plos One (23,774 times), Critical Care (21,499 times), JAMA (20,633 times) and Journal of Clinical Investigation (20,633 times). Therefore, Critical Care Medicine was the predominant journal in sepsis associated with ALI globally according to co-citation analysis.

**TABLE 6:** The top 10 co-citation journals.

No.	Journals	Citations	Total link strength	If	JCR
1	Critical Care Medicine	1,503	52,752	9.296	Q1
2	American Journal of Respiratory and Critical Care Medicine	1,196	46,606	30.582	Q1
3	New England Journal of Medicine	920	36,234	176.09	Q1
4	Journal of Immunology	805	34,933	5.426	Q2
5	American Journal of Physiology-lung Cellular and Molecular Physiology	858	33,680	6.011	Q1/Q2
6	Shock	730	23,489	3.533	Q2/Q3
7	Plos One	531	23,774	3.752	Q2
8	Critical Care	581	21,499	19.334	Q1
9	JAMA-Journal of The American Medical Association	533	20,633	157.335	Q1
10	Journal of Clinical Investigation	497	20,633	19.456	Q1

#### 3.7.2 Authors

**FIGURE 5:**
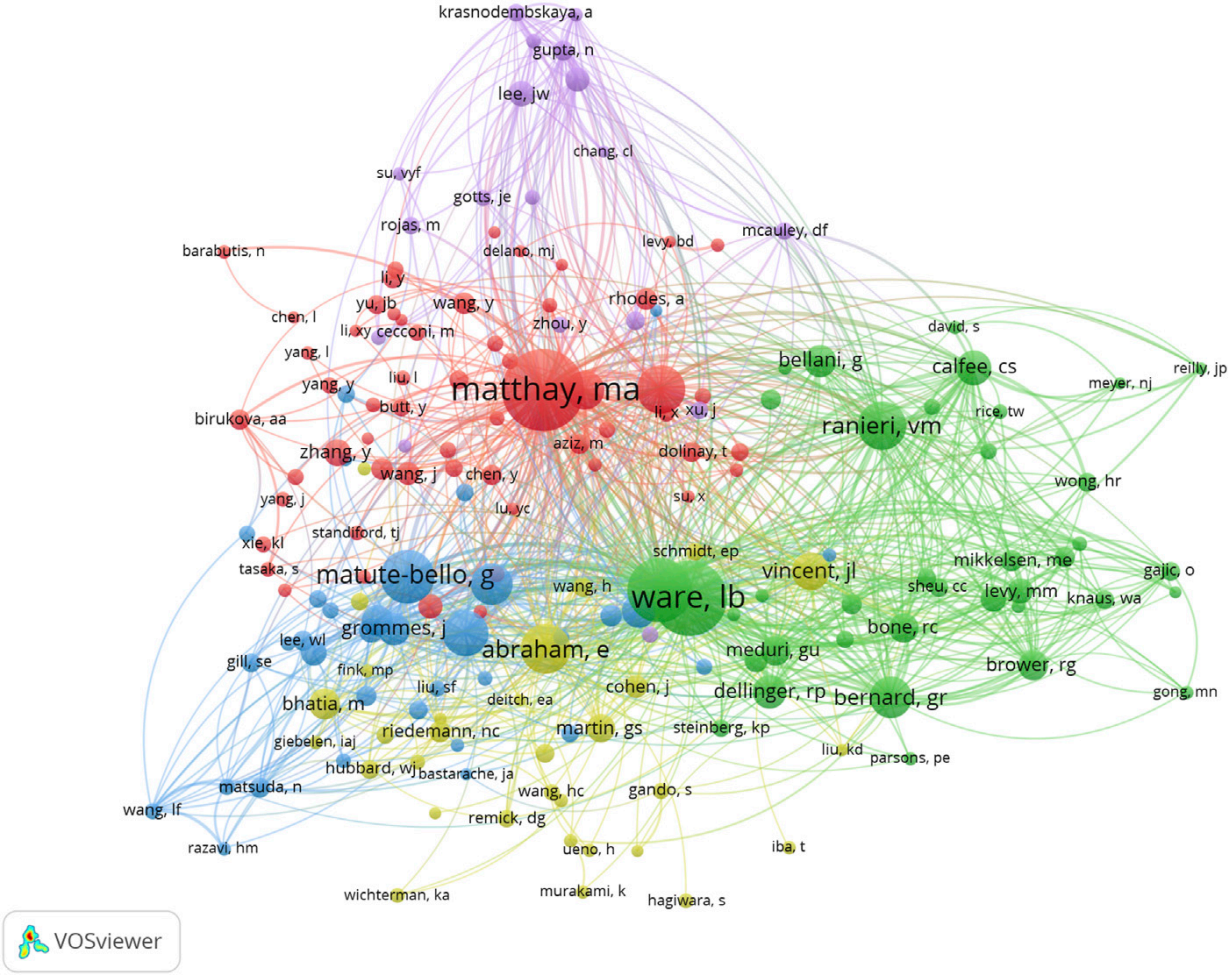
The mapping on authors of sepsis associated with ALI. The relationship between 179 identified authors (the minimum number of citations of journals exceeds 15) and the total link strength.

Figure 5 shows the relationship between 179 identified authors (the minimum number of citations of journals exceeds 15) and the total link strength using VOSviewer. As presented in Table 7, the top 10 authors by total link strength were as follows: Matthay MA (total link strength = 1,753 times), Ware LB (1,439 times), Rubenfeld GD (1,239 times), Abraham E (935 times), Ranieri VM (933 times), Matute-Bello G (780 times), Singer M (768 times), Calfee CS (742 times), Bernard GR (714 times) and Hotchkiss RS (666 times). Therefore, Matthay MA was the predominant author in sepsis associated with ALI globally according to co-citation analysis.

**TABLE 7:** The top 10 co-citation authors.

No.	Authors	Citations	Total link strength	Country
1	Matthay MA	251	1,753	United States of America
2	Ware LB	218	1,439	United States of America
3	Rubenfeld GD	174	1,236	Canada
4	Abraham E	116	935	United States of America
5	Ranieri VM	113	933	Italy
6	Matute-bello G	132	780	United States of America
7	Singer M	111	768	United Kingdom
8	Calfee CS	70	742	United States of America
9	Bernard GR	92	714	United States of America
10	Hotchkiss RS	89	666	United States of America

#### 3.7.3 References

**FIGURE 6:**
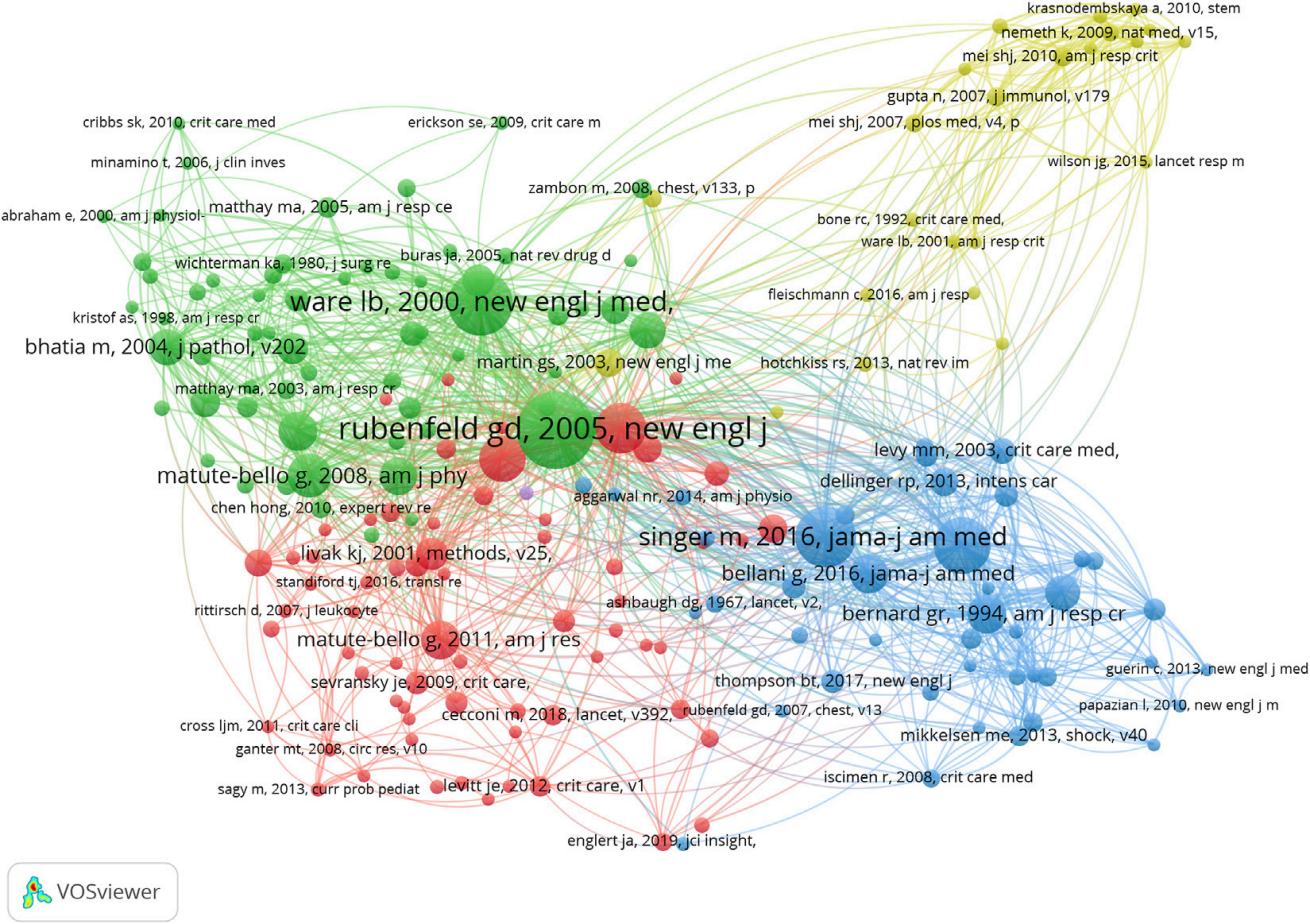
The mapping on references of sepsis associated with ALI. The relationship between 193 identified references (the minimum number of citations of journals exceeds 10) and the total link strength.

Figure 6 shows the relationship between 193 identified references (the minimum number of citations of journals exceeds 10) and the total link strength using VOSviewer. As presented in Table 8, the top 10 references by total link strength were as follows: Rubenfeld GD, “Incidence and outcomes of acute lung injury (Rubenfeld et al., 2005)”, New England Journal of Medicine, 2005 (total link strength = 932 times). Ware LB, “The acute respiratory distress syndrome (Ware and Matthay, 2000)”, New England Journal of Medicine, 2000 (602 times). Ranieri VM, “Acute respiratory distress syndrome: the Berlin Definition (Ranieri et al., 2012)”, JAMA, 2012 (581 times). Singer M, “The Third International Consensus Definitions for Sepsis and Septic Shock (Sepsis-3) (Singer et al., 2016)”, JAMA, 2016 (579 times). Matthay MA, “The acute respiratory distress syndrome (Matthay et al., 2012)”, Journal of Clinical Investigation, 2012 (495 times). Grommes J, “Contribution of neutrophils to acute lung injury (Grommes and Soehnlein, 2011)”, Molecular Medicine, 2011 (395 times). Rittirsch D, “Immunodesign of experimental sepsis by cecal ligation and puncture (Rittirsch et al., 2009)”, Nature Protocols, 2009 (381 times). Matute-Bello G, “Animal models of acute lung injury (Matute-Bello et al., 2008)”, American Journal of Physiology-Lung Cellular, 2008 (364 times). Bellani G, “Epidemiology, Patterns of Care, and Mortality for Patients With Acute Respiratory Distress Syndrome in Intensive Care Units in 50 Countries (Bellani et al., 2016)”, JAMA, 2016 (338 times) and Bernard G, “The American-European Consensus Conference on ARDS. Definitions, mechanisms, relevant outcomes, and clinical trial coordination (Bernard et al., 1994)”, American Journal of Respiratory and Critical Care Medicine, 1994 (320 times). Therefore, “Incidence and outcomes of acute lung injury (Rubenfeld et al., 2005)” was the predominant reference in sepsis associated with ALI globally according to co-citation analysis. The authors of this literature conducted a prospective, population-based cohort study at 21 hospitals using a validated screening protocol to identify patients meeting consensus criteria for ALI.

**TABLE 8:** The top 10 co-citation references.

No.	Title	Times cited	Total link strength	Author	Journal	Year	Type	If (2021)
1	Incidence and outcomes of acute lung injury	153	932	Rubenfeld GD et al	New England Journal of Medicine	2005	Article	176.079
2	The acute respiratory distress syndrome	117	602	Ware LB et al	New England Journal of Medicine	2000	Review	176.079
3	Acute respiratory distress syndrome: the Berlin Definition	97	581	Ranieri VM et al	JAMA-Journal of The American Medical Association	2012	Article	157.335
4	The Third International Consensus Definitions for Sepsis and Septic Shock (Sepsis-3)	107	579	Singer M et al	JAMA-Journal of The American Medical Association	2016	Article	157.335
5	The acute respiratory distress syndrome	80	495	Matthay MA et al	Journal of Clinical Investigation	2012	Review	19.456
6	Contribution of neutrophils to acute lung injury	54	395	Grommes J et al	Molecular Medicine	2011	Review	6.376
7	Immunodesign of experimental sepsis by cecal ligation and puncture	71	381	Rittirsch D et al	Nature Protocols	2009	Article	17.021
8	Animal models of acute lung injury	64	364	Matute-Bello G et al	American Journal of Physiology-Lung Cellular and Molecular Physiology	2008	Review	6.011
9	Epidemiology, Patterns of Care, and Mortality for Patients With Acute Respiratory Distress Syndrome in Intensive Care Units in 50 Countries	53	338	Bellani G et al	JAMA-Journal of The American Medical Association	2016	Article	157.335
10	The American-European Consensus Conference on ARDS. Definitions, mechanisms, relevant outcomes, and clinical trial coordination	55	320	Bernard G R et al	American Journal of Respiratory and Critical Care Medicine	1994	Review	30.528

### 3.8 The analysis of keywords

**FIGURE 7:**
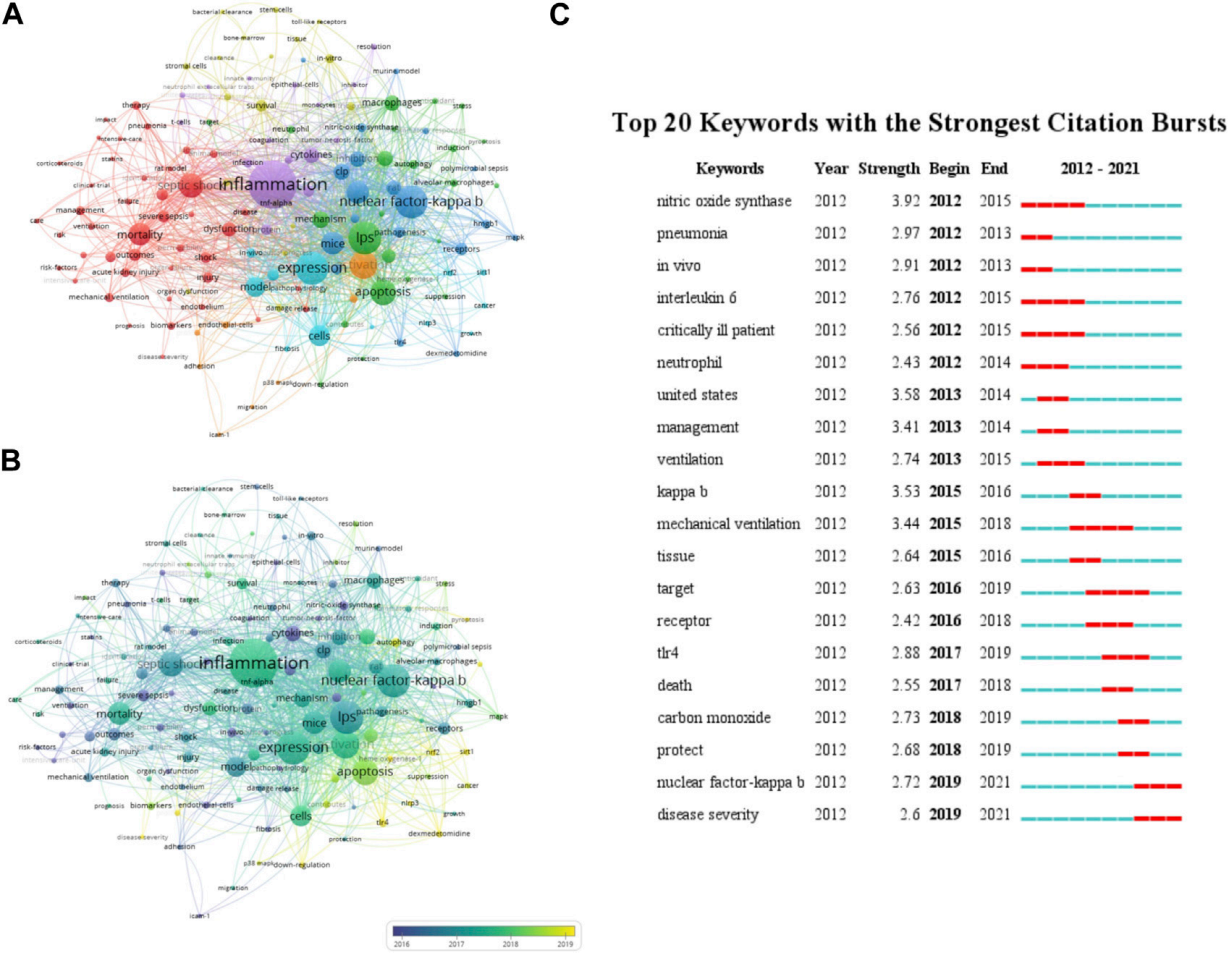
The mapping on keywords of sepsis associated with ALI **(A)** Visualization of 151 identified keywords in sepsis associated with ALI; **(B)** Overlay visualization of the 151 identified keywords in sepsis associated with ALI based on the average time they appeared in the publications. The blue keyword appeared earlier, while the yellow keyword appeared later. **(C)** The top 25 terms with the most cited bursts.

Keywords usually reflect the theme of research, and systematic analysis of keywords reflect the research hotspot in a specific field. When analyzing the keywords, we combined some different versions of the same terms, including different spelling versions (e.g., “nf-kappa-b”, “nuclear factor-kappa b” and “NF-κB”), and abbreviated terms (e.g., “LPS” and “lipopolysaccharide”). Table 9 shows the top 20 keywords, with most of them falling into two groups. One group is mechanisms involved in sepsis associated with ALI, such as inflammation, NF-κB, cytokines and oxidative stress. The other group is research methods involved in sepsis associated with ALI, including keywords like LPS (lipopolysaccharide), CLP (cecal ligation and puncture), and macrophages. In Figure 7A, we used VOSviewer software to show the primary keywords more compact. As presented in Figure 7B, the blue keywords appeared earlier, while the yellow keywords appeared later. The results of co-occurrence analysis indicated that programmed cell death (PCD) might become the hot spot of future sepsis associated with ALI research. The evolution of a knowledge domain can be represented by citation bursts. Citation burst refers to the literature that has attracted the attention of scholars in a specific field during a specific period of time. CiteSpace has a burst detection function, which we use to find the top 25 terms with the most cited bursts (Figure 7C). Keywords related to ALI, such as nitric oxide synthase, pneumonia, and interleukin-6, began to burst at an early stage. Subsequently, keywords such as mechanical ventilation began to burst, indicating that the focus of the study has shifted to the treatment of ALI. It is also noteworthy that TLR4 and NF-κB only had bursts recently.

**TABLE 9:** The top 20 Keywords.

No.	Keywords	Count
1	inflammation	289
2	lps	164
3	nuclear factor-kappa b	164
4	expression	161
5	activation	126
6	septic shock	106
7	mice	94
8	oxidative stress	92
9	mortality	91
10	cells	80
11	model	77
12	mechanism	59
13	clp	57
14	cytokines	54
15	inhibition	54
16	pathway	53
17	macrophages	52
18	rat	49
19	dysfunction	47
20	protects	42

## 4 Discussion

Bibliometrics plays a very important role in improving the ability of literature retrieval and utilization, helping beginners to rapidly enter a particular field. For the first time, we reported the bibliometric analysis to review the progress of sepsis associated with ALI related research in worldwide in the past decade. At present, research on the sepsis associated with ALI is flourishing. In the initial phase, the definition of ALI and diagnostic criteria predominated and, nowadays, the focus of interest is on understanding the pathophysiological mechanisms that lead to apoptosis, necroptosis and pyroptosis. The research on programmed cell death is a hot spot and may be a promising research field in the coming years.

Among the top 10 countries, China ranked first in the total number of publications, cited times and H-index, which indicated that China is a high-yield country in the study of sepsis associated with ALI, and the United States ranked first in average number of citations per publication, which means that the United States has more influence in this field. Seven Chinese affiliations came in the top 10 affiliations in the research of sepsis associated with ALI, the United States affiliations only three came in the top 10 affiliations, but the United States affiliations had a relatively high cited times, average number of citations per publication and H-index. Among the top journals, International Immunopharmacology ranked first in the sum of total publications, Inflammation first in H-index, Critical Care ranked first in cited times, average number of citations per publication and IF. Six authors of the United States came in the top 10 authors in the research of sepsis associated with ALI, Chinese and Spain each have two authors came in the top 10 authors. Matthay MA has published the most papers. His important contribution to this field is he screened a number of markers and therapeutic targets related to sepsis associated with ALI through clinical and basic research. Although China ranked first in the sum of total publications, only two authors have come in the top 10 authors in the world, indicating that China still needs to increase investment in this field to lead the research trend. Among the top 10 most cited publications, “The pulmonary endothelial glycocalyx regulates neutrophil adhesion and lung injury during experimental sepsis” ranked first, which was published in Nature Medicine in 2012 and has been cited 439 times in total. The article found that heparinase inhibition could alleviating sepsis-induced ALI and mortality in mice. From the perspective of co-cited journals, Critical Care Medicine had been the most co-cited journal. From the perspective of co-cited authors, Matthay MA had been the most co-cited author. Among the 24,236 co-cited references retrieved, Table 8 shows the top 10 references by total link strength, of which “Incidence and outcomes of acute lung injury” is the most frequently cited.

**FIGURE 8:**
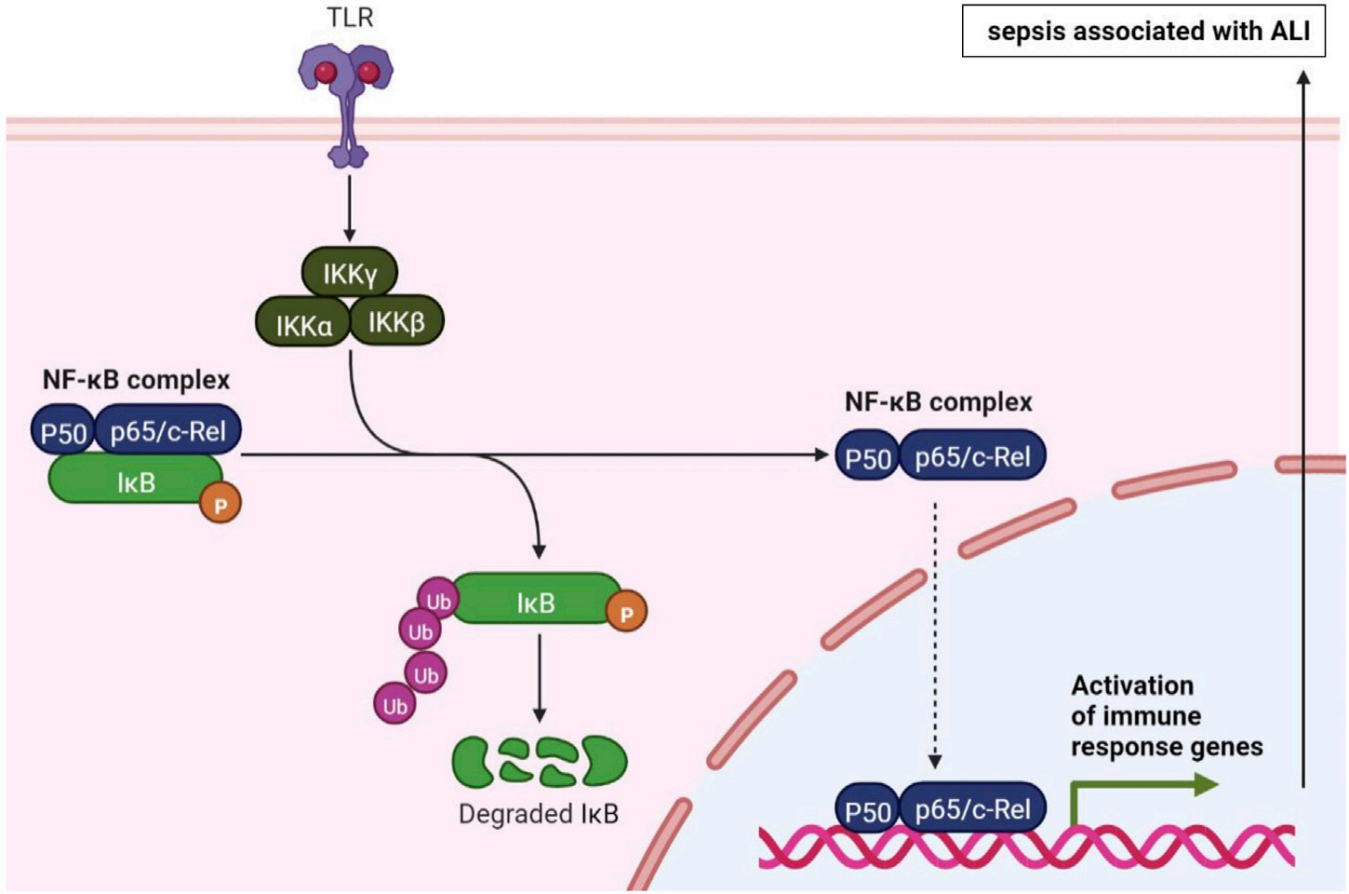
The mechanism of NF-κB in sepsis associated with ALI (Created with BioRender.com).

In the results, a systematic bibliometric analysis has been conducted on the studies on sepsis associated with ALI published in WOS from 2012 to 2021. Table 9 shows the top 20 keywords, with most of them falling into two groups. One group is mechanisms involved in sepsis associated with ALI, such as inflammation, NF-κB, oxidative stress and cytokines. The other group is research methods involved in sepsis associated with ALI, including keywords like LPS, CLP, and macrophages. NF-κB is an important nuclear transcription factor in cells. It is related to the inflammatory changes of many human diseases, such as ALI, rheumatoid arthritis, heart and brain diseases (Gong et al., 2021; Jiao et al., 2021; Wang et al., 2021; Xie et al., 2022). Many studies have demonstrated that NF-κB pathways are involved in sepsis associated with ALI (Wang et al., 2014; Li et al., 2016; Wang et al., 2020; Du et al., 2021). There are five members of NF-κB family, including RelA (p65), RelB, NF-κB1 (P50), NF-κB2 (P52) and c-Rel (Jimi and Katagiri, 2022). Commonly referred to as NF-κB protein, it refers to NF-κB1 dimer protein formed by P65/p50 subunit. The classical NF-κB pathway can be activated by various stimuli (e.g., TNF-α, LPS, IL-1β). It is mediated by cell surface receptors, such as TLR, TNF-R and antigen receptors, and activated by various adaptor proteins and signaling kinase IKK (including IKKα, IKKβ and IKKγ). Activation of the IKK complex leads to phosphorylation of the NF-κB inhibitor protein IκBα, which ultimately leads to proteasome degradation and release and translocation of NF-κB dimers, such as p50-RelA (p65), to the nucleus to drive transcription of target genes. The activation of immune response genes ultimately leads to sepsis associated with ALI (Figure 8).

The results of co-occurrence analysis suggested that PCD might become a research hotspot of sepsis associated with ALI in the future, and before 2017, most studies focused on the study of complications and inflammation. PCD is a genetically regulated process leading to the death of cells. It plays a critical role in organismal development, homeostasis, and pathogenesis (Liang and He, 2022). At present, it is found that besides apoptosis, PCD also includes necroptosis and pyroptosis. According to our study, PCD has been extensively involved in sepsis associated with ALI studies in recent years.

**FIGURE 9:**
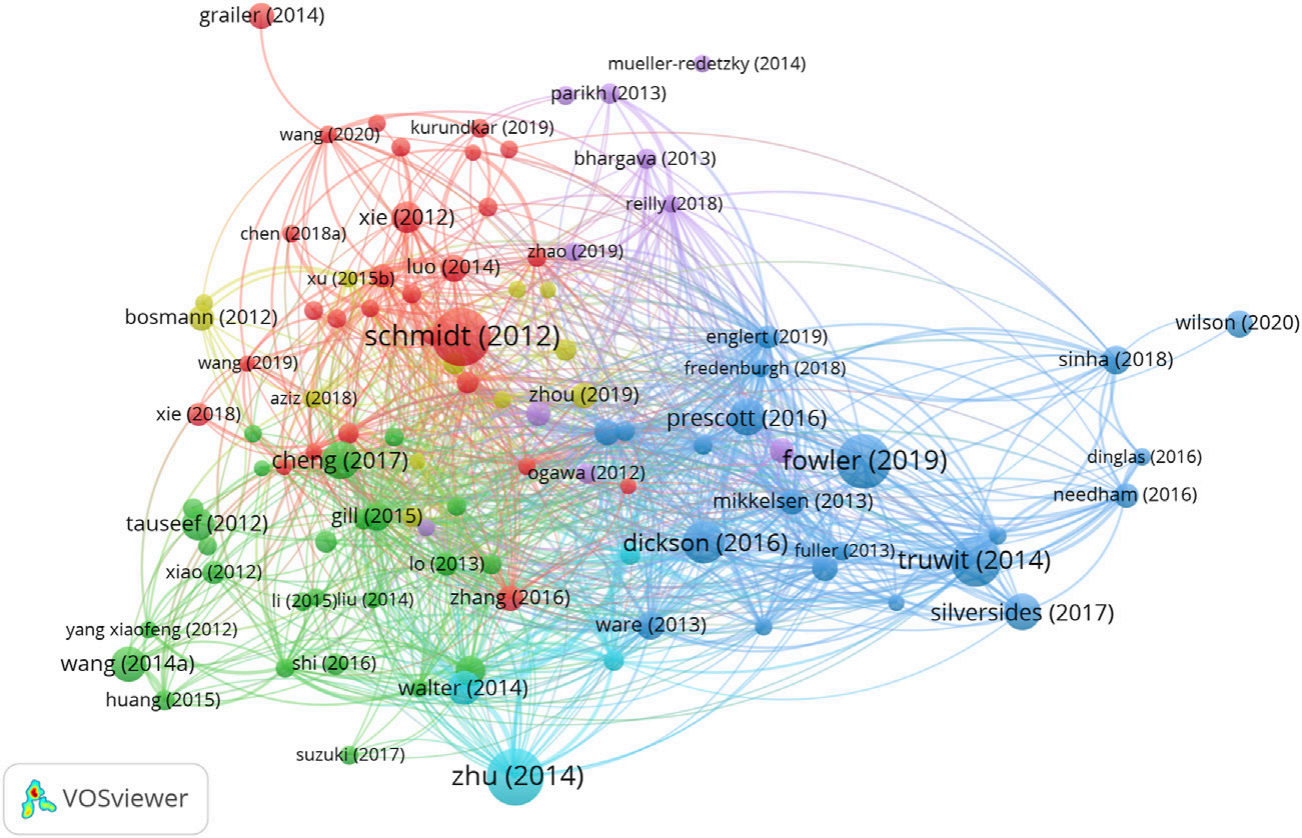
The mapping on top 100 cited references of sepsis associated with ALI.

It is important to clarify the future research direction of sepsis associated with ALI. Therefore, the recent articles with high citation rate should be divided into subgroups to analyze their research hotspots. As presented in Figure 9, the top 100 cited articles were used for bibliographic coupling and divided into 6 clusters according to the research direction. Cluster #1 contains 28 articles, the main research topic is the effect of different drugs on sepsis associated with ALI. Schmidt EP et al., in 2012 found that heparanase inhibition prevented endotoxemia-associated glycocalyx loss and neutrophil adhesion and, accordingly, attenuated sepsis-induced ALI and mortality in mice. Cluster #2 contains 25 articles, the main research topic is the pathogenesis of sepsis associated with ALI. Cheng KT et al., in 2017 discovered that caspase-11-mediated endothelial pyroptosis underlies sepsis-induced ALI (Cheng et al., 2017). Toll-like receptor 4 (TLR4) activation of TRPC6-dependent calcium signaling mediates endotoxin-induced lung vascular permeability and inflammation (Tauseef et al., 2012). Cluster #3 contains 21 articles, the main research topic is clinical study of sepsis associated with ALI. In CITRIS-ALI randomized clinical trial, a 96-h infusion of vitamin C compared with placebo did not significantly improve organ dysfunction scores or alter markers of inflammation and vascular injury (Fowler et al., 2019). Many common drugs, including statins (Mansur et al., 2015; Dinglas et al., 2016; Needham et al., 2016), hydrocortisone (Tongyoo et al., 2016), carbon monoxide (Fredenburgh et al., 2018) and aspirin (Toner et al., 2015), have been tested in clinical trials for the treatment of sepsis-induced ALI. Cluster #4 contains 12 articles, the main research topic is the role of intracellular substances in sepsis associated with ALI. Zhou et al., in 2019 demonstrated that human endothelial progenitor cell (EPC) exosomes are beneficial in LPS-induced ALI mice (Zhou et al., 2019). Jiang J et al. identified that targeting NOX4 may be an innovative therapeutic option that is markedly effective in treating sepsis-induced ALI (Jiang et al., 2020). Cluster #5 contains 10 articles, the main research topic is markers and therapeutic targets of sepsis associated with ALI. Reilly JP et al. found that plasma angiopoietin-2 as a potential causal marker in sepsis-associated ALI development (Reilly et al., 2018). Cluster #6 contains 4 articles, the main research topic is application of mesenchymal stem cells (MSC) in sepsis associated with ALI. Human MSC-derived microvesicles were therapeutically effective following *E. coli* sepsis-induced ALI in mice (Zhu et al., 2014).

## 5 Limitations

There are some limitations in the present study. First of all, we chose WOS as the only literature search database, which may have missed some publications, resulting in database bias. Second, only literatures published during 2012–2021 were included, articles published before were missed. Finally, some of the latest high-quality articles may not be enrolled in top 10 journals and top 10 most cited publications due to the low number of citations. However, we believe that this work can still be used to present the overall situation and trends in this field.

## 6 Conclusion

China is a major producing country, and the United States has more influence in this field. Shanghai Jiao Tong University, University of California System and Huazhong University of Science Technology were the main contributing institutions to sepsis associated with ALI. International Immunopharmacology, Inflammation, Shock and Critical Care have published the latest studies and novel progress in this field. Matthay MA and Ware LB were the main contributors to this field. Inflammation and NF-κB have always been the focus of sepsis associated with ALI related research, and PCD (including apoptosis, necroptosis and pyroptosis) may be the important direction of future research. Overall, our study will provide useful reference for further research on sepsis associated with ALI.

## Data Availability

The raw data supporting the conclusions of this article will be made available by the authors, without undue reservation.
